# Pharmacokinetics of high-titer anti–SARS-CoV-2 human convalescent plasma in high-risk children

**DOI:** 10.1172/jci.insight.151518

**Published:** 2022-01-25

**Authors:** Oren Gordon, Mary Katherine Brosnan, Steve Yoon, Dawoon Jung, Kirsten Littlefield, Abhinaya Ganesan, Christopher A. Caputo, Maggie Li, William R. Morgenlander, Stephanie N. Henson, Alvaro A. Ordonez, Patricia De Jesus, Elizabeth W. Tucker, Nadine Peart Akindele, Zexu Ma, Jo Wilson, Camilo A. Ruiz-Bedoya, M. Elizabeth M. Younger, Evan M. Bloch, Shmuel Shoham, David Sullivan, Aaron A.R. Tobian, Kenneth R. Cooke, Ben Larman, Jogarao V.S. Gobburu, Arturo Casadevall, Andrew Pekosz, Howard M. Lederman, Sabra L. Klein, Sanjay K. Jain

**Affiliations:** 1 Division of Infectious Diseases, Department of Pediatrics, Johns Hopkins University School of Medicine, Baltimore, Maryland, USA.; 2 W. Harry Feinstone Department of Molecular Microbiology and Immunology, Johns Hopkins University Bloomberg School of Public Health, Baltimore, Maryland, USA.; 3 Center for Translational Medicine, University of Maryland School of Pharmacy, Baltimore, Maryland, USA.; 4 Division of Immunology, Department of Pathology,; 5 Department of Anesthesiology and Critical Care Medicine,; 6 Division of Immunology, Department of Pediatrics,; 7 Division of Transfusion Medicine, Department of Pathology,; 8 Division of Infectious Diseases, Department of Medicine, and; 9 Department of Oncology, Sidney Kimmel Comprehensive Cancer Center, Johns Hopkins University School of Medicine, Baltimore, Maryland, USA.

**Keywords:** COVID-19, Infectious disease, Immunoglobulins

## Abstract

**BACKGROUND:**

While most children who contract COVID-19 experience mild disease, high-risk children with underlying conditions may develop severe disease, requiring interventions. Kinetics of antibodies transferred via COVID-19 convalescent plasma early in disease have not been characterized.

**METHODS:**

In this study, high-risk children were prospectively enrolled to receive high-titer COVID-19 convalescent plasma (>1:320 anti-spike IgG; Euroimmun). Passive transfer of antibodies and endogenous antibody production were serially evaluated for up to 2 months after transfusion. Commercial and research ELISA assays, virus neutralization assays, high-throughput phage-display assay utilizing a coronavirus epitope library, and pharmacokinetic analyses were performed.

**RESULTS:**

Fourteen high-risk children (median age, 7.5 years) received high-titer COVID-19 convalescent plasma, 9 children within 5 days (range, 2–7 days) of symptom onset and 5 children within 4 days (range, 3–5 days) after exposure to SARS-CoV-2. There were no serious adverse events related to transfusion. Antibodies against SARS-CoV-2 were transferred from the donor to the recipient, but antibody titers declined by 14–21 days, with a 15.1-day half-life for spike protein IgG. Donor plasma had significant neutralization capacity, which was transferred to the recipient. However, as early as 30 minutes after transfusion, recipient plasma neutralization titers were 6.2% (range, 5.9%–6.7%) of donor titers.

**CONCLUSION:**

Convalescent plasma transfused to high-risk children appears to be safe, with expected antibody kinetics, regardless of weight or age. However, current use of convalescent plasma in high-risk children achieves neutralizing capacity, which may protect against severe disease but is unlikely to provide lasting protection.

**Trial registration:**

ClinicalTrials.gov NCT04377672.

**Funding:**

The state of Maryland, Bloomberg Philanthropies, and the NIH (grants R01-AI153349, R01-AI145435-A1, K08-AI139371-A1, and T32-AI052071).

## Graphical abstract

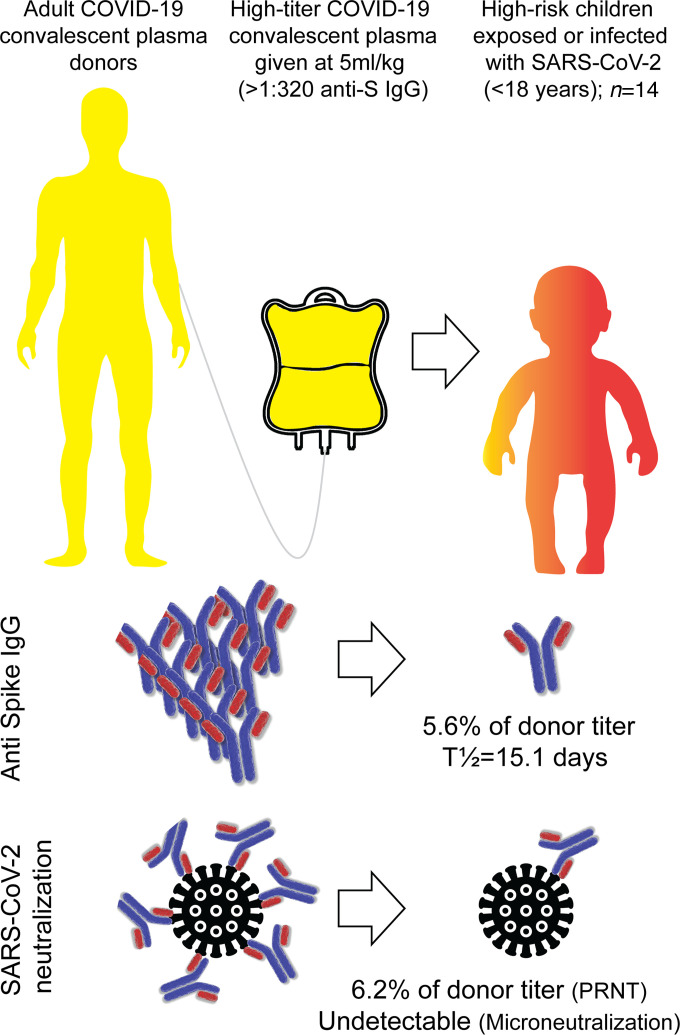

## Introduction

Passive transfer of immunity (e.g., antibody therapy) has been used to prevent and treat infectious diseases (1, 2). Immunoglobulins are routinely used in clinical practice to prevent infections, such as rabies, hepatitis B, chickenpox, and respiratory syncytial virus, in exposed, susceptible children (3). Similarly, passive transfer of maternal antibodies renders protection against several pathogens in infants (4). Coronavirus disease 2019 (COVID-19), which is caused by the severe acute respiratory syndrome coronavirus 2 (SARS-CoV-2), is usually mild in children. Some children, however, may have higher risk of disease progression due to underlying conditions (5–9). The role of antibody-based therapeutics for COVID-19 is being investigated, and data suggest that early administration of either high-titer convalescent plasma or neutralizing monoclonal antibodies may be useful (10–12). Randomized controlled trials to evaluate COVID-19 convalescent plasma for prophylaxis and early treatment in adults are currently underway (13, 14). Studies evaluating antibody kinetics of convalescent plasma have been conducted in infected individuals who developed endogenous anti–SARS-CoV-2 antibodies (15). However, antibody kinetics in SARS-CoV-2–exposed individuals or those early in disease and without endogenous antibody production, where antibody therapies are most useful (11), have not been well characterized. Additionally, characteristics such as safety and antibody pharmacokinetics may also differ in children.

**Figure 1:**
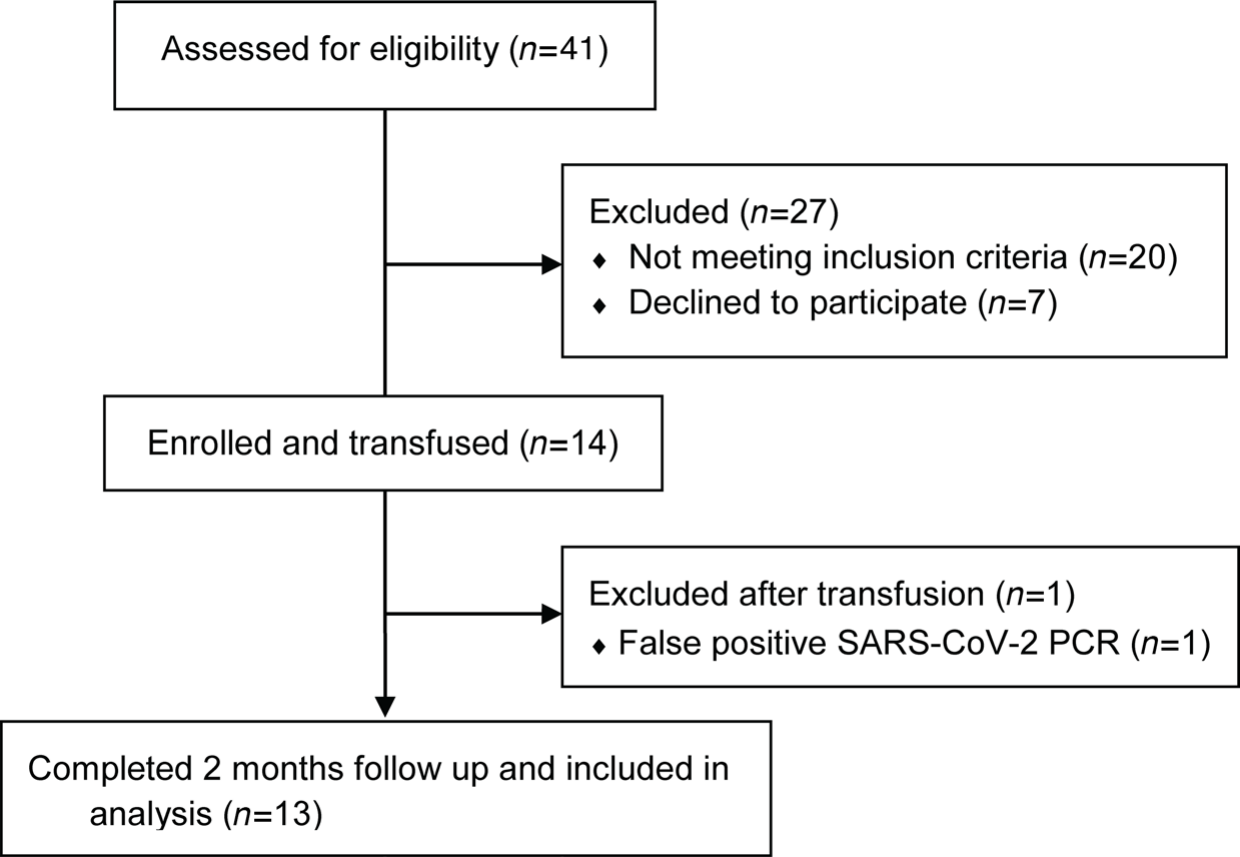
Study flow diagram.

This study (ClinicalTrials.gov NCT04377672), conducted at Johns Hopkins Children’s Center (between May 2020 and April 2021; see Methods, Figure 1, and Supplemental Figure 1; supplemental material available online with this article; https://doi.org/10.1172/jci.insight.151518DS1), was performed under a US FDA exploratory investigational new drug application. High-risk children exposed to or infected with SARS-CoV-2 were enrolled prospectively and received pretitered COVID-19 convalescent plasma. Passive transfer of antibodies and endogenous antibody production were evaluated in study participants serially for up to 2 months after plasma administration. Several antibody tests, including 2 commercial assays, research ELISAs, viral neutralization assays, a high-throughput phage-display assay utilizing a coronavirus epitope library, and pharmacokinetic analyses, were performed.

## Results

Fourteen high-risk children (8 female and 6 male children; median age, 7.5 years; range, 3 months to 17 years) were prospectively enrolled and received high-titer COVID-19 convalescent plasma (>1:320 anti-spike [anti-S] IgG by Euroimmun assay corresponding to >1:1620 anti-S IgG using a research ELISA) (Figure 1 and Table 1). Detailed clinical and laboratory data for all participants are outlined in Supplemental Tables 1–4. Nine children received early treatment within 5 days (range, 2–7 days) of symptom onset and 5 received prophylaxis within 4 days (range, 3–5 days) after exposure to SARS-CoV-2. There were no serious adverse events (AEs) related to plasma administration. Three participants developed a rash that resolved rapidly and with no sequelae. One participant was excluded after receiving plasma, due to a false-positive SARS-CoV-2 test. Passive transfer of antibodies and endogenous antibody production were evaluated in all recipients for up to 2 months after plasma administration.

**Figure 2:**
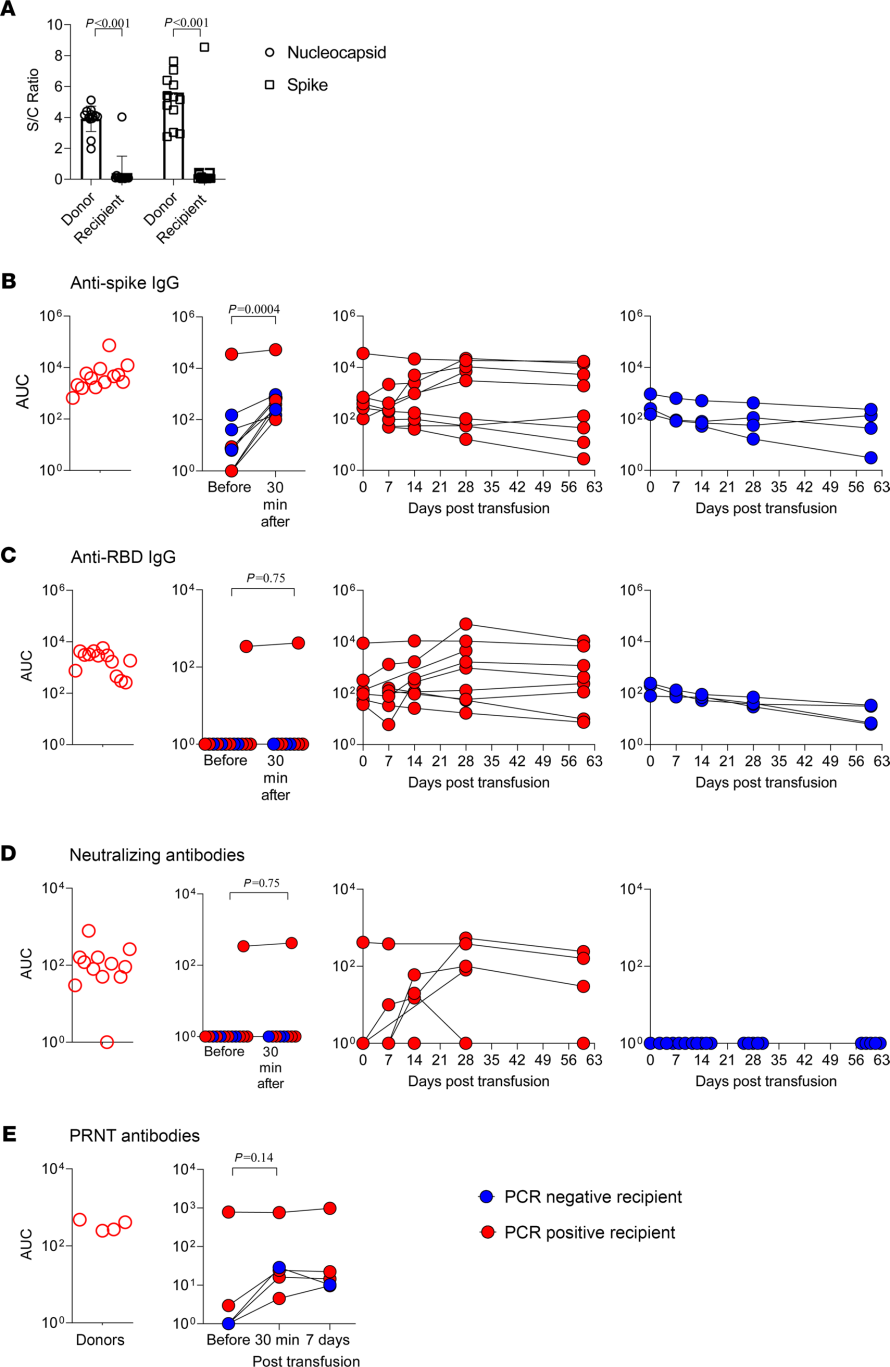
SARS-CoV-2 antibody pharmacokinetics and neutralization capacity in donors and recipients of COVID-19 convalescent plasma. (**A**) Commercial ELISA assays were used to measure SARS-CoV-2 anti-nucleocapsid protein IgG/IgM/IgA (Bio-Rad) and anti-spike IgG (Euroimmun) titers of donor and recipient plasma prior to plasma administration (*n* = 13 pairs of donors and recipients). S/C, signal-to-cutoff ratio. (**B** and **C**) Indirect ELISAs were used to measure IgG antibody levels against spike or spike receptor binding domain (RBD) in 3-fold serial dilution starting at 1:20. Results are presented as AUC values on a logarithmic scale (*n* = 12 donors and *n* = 13 recipients for all time points other than 30 minutes, for which *n* = 9). (**D**) Microneutralization assay was performed on each plasma sample in 2-fold serial dilutions starting at 1:20. Results are presented as AUC values on a logarithmic scale (*n* = 12 donors and *n* = 13 recipients for all time points other than 30 minutes, for which *n* = 9). (**E**) Plaque reduction neutralization test (PRNT_50_) was performed on plasma from 5 recipients (recipients 9–13) and 4 donors (donors 9 and 11–13) in 2-fold serial dilutions starting at 1:10. Results are presented as AUC values on a logarithmic scale. A 2-tailed Mann-Whitney *U* test was used to compare donor to recipient titers and recipient titers before and 30 minutes after transfusion. The corresponding *P* values are shown.

SARS-CoV-2 anti-nucleocapsid and anti-S antibodies were undetectable in all recipients before plasma administration (as measured by the Bio-Rad and Euroimmun commercial ELISA assays, respectively), except for recipient 13 (Figure 2A). The donor geometric mean and range for anti–receptor binding domain (anti-RBD) and anti-S titers were 1:2478 (1:180 to 1:14,580) and 1:6271 (1:1,620 to 1:393,660), respectively (Figure 2, B and C). Recipient titers 30 minutes after plasma transfusion (available for 9 participants, participants 5–13) were on average 5.6% (range, 1.2%–7.5%) of donor titers. Recipient antibody titers declined by 14–21 days (Figure 2, B and C), although 7 SARS-CoV-2–infected participants demonstrated an increase due to endogenous antibody production (participants 1, 4, 6, 7, and 11–13). Three children who developed severe pneumonia requiring positive pressure ventilation (participants 6, 7, and 11) had the highest endogenous antibody titers (Figure 2, B and C). While serology using commercial assays (Euroimmun and Bio-Rad) yielded positive results for all the donors, they were positive in only 5 recipients (recipients 6, 7, and 11–13) who also had the highest endogenous antibody titers (Supplemental Figure 2).

Pharmacokinetic analysis was performed in participants without endogenous antibody production within the first 14 days after transfusion (*n* = 7; Supplemental Figure 3). The mean half-lives were estimated to be 15.1 and 15.4 days for anti-S and anti-RBD IgG, respectively, and were consistent with the expected half-life for IgG (16). The clearance and half-life were independent of body weight or age.

All donor plasma (but that from one donor) had neutralization capacity, as measured by the microneutralization assay, with a geometric mean titer of 1:97 (range 1:40 to 1:640; Figure 2D). However, recipient plasma (even as early as 30 minutes after transfusion) had undetectable neutralization capacity using this assay, unless endogenous antibody responses developed (Figure 2D). Results of a plaque reduction neutralization test (PRNT_50_) were available for 5 recipients (recipients 9–13) and 4 donors (donors 9 and 11–13). Recipient neutralization titers 30 minutes after plasma transfusion were on average 6.2% of donor titer (range, 5.9%–6.7%) and declined to 4.5% (range, 2.1%–5.8%) by 7 days after transfusion or otherwise increased due to endogenous immune response (Figure 2E).

**Figure 3:**
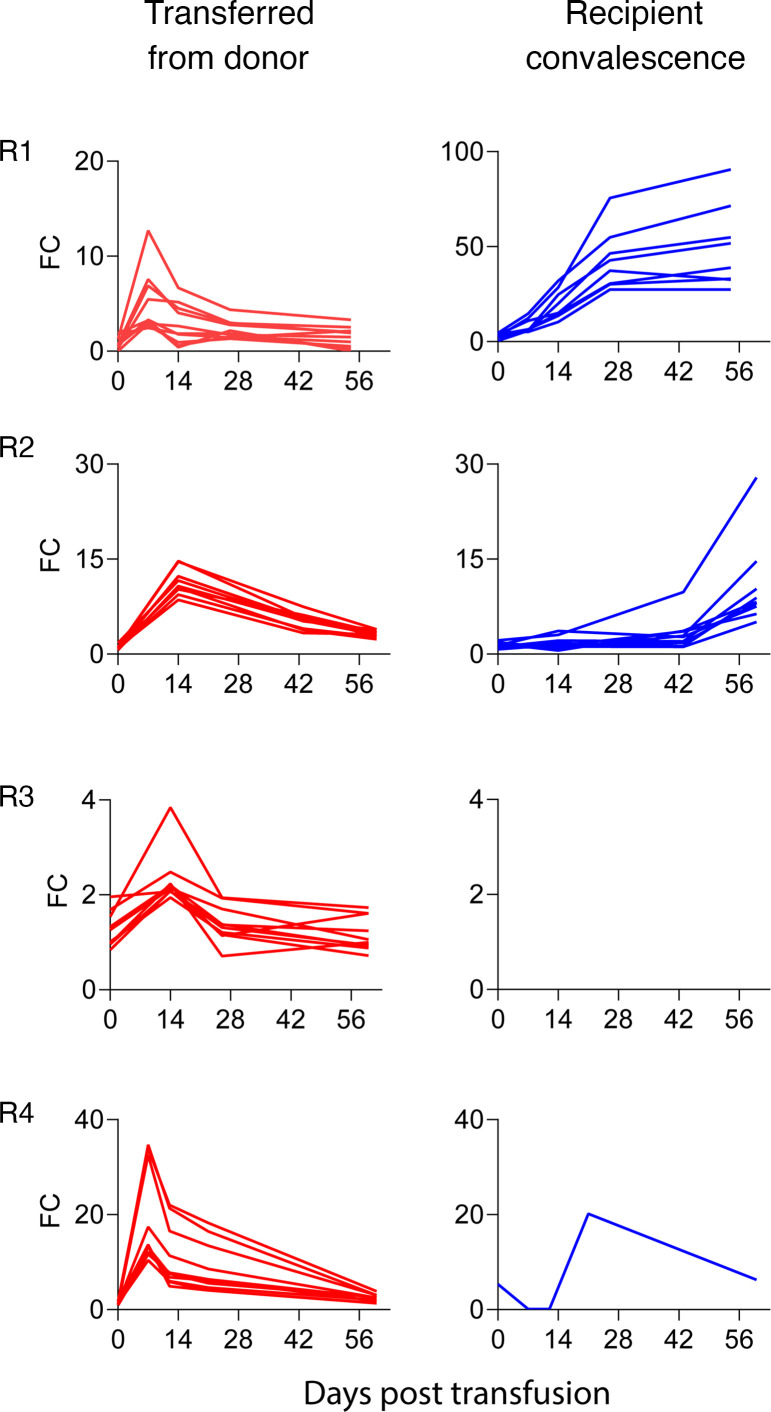
Kinetics of transferred and endogenous pan-coronavirus antibodies. Plasma from 4 recipients (R1–R4) was subjected to VirScan, a phage-display library of epitopes from various coronaviruses, including SARS-CoV-2. Fold change (FC) was measured for antibody subsets in recipient plasma (each line represents a single antibody subset): donor antibodies (red) and endogenous antibodies (blue). Note that participants R1, R2, and R4, who developed SARS-CoV-2 infection, demonstrate endogenous antibody production. However, participant R3, who remained asymptomatic and SARS-CoV-2 PCR negative throughout the study period, did not develop an endogenous response.

**Figure 4:**
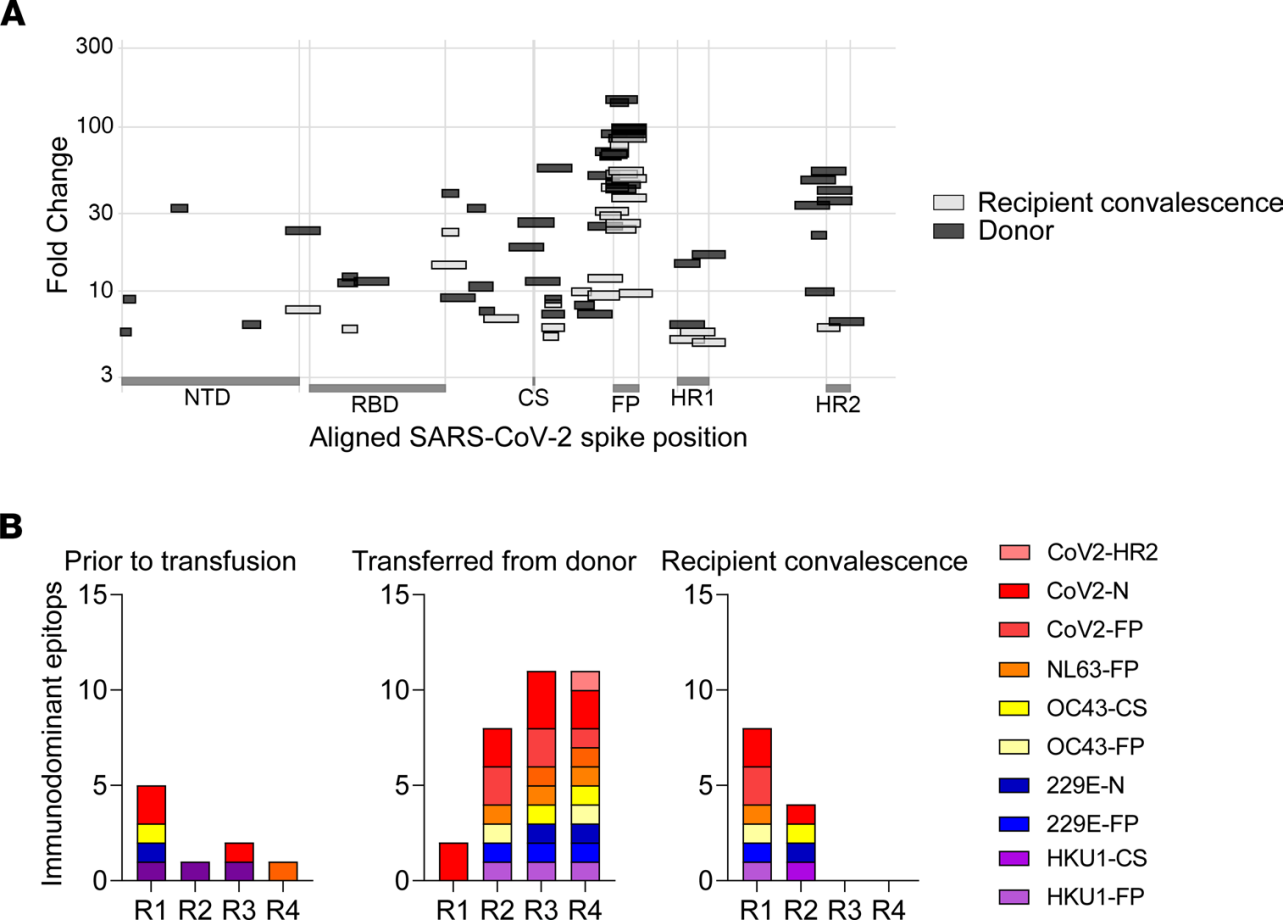
Transfer of pan-coronavirus immunodominant antibodies. Plasma from 4 donor-recipient pairs (R1–R4) were subjected to VirScan, a phage-display library of epitopes from various coronaviruses, including SARS-CoV-2 and the 4 endemic coronaviruses (NL63, OC43, 229E, and HKU1). (**A**) Donor and recipient convalescent antibody reactivities to SARS-CoV-2 spike protein. (**B**) The number of antibodies targeting pan-coronavirus immunodominant epitopes is presented for each group. Only antibodies unique to each group are shown. Immunodominant epitopes were previously defined (17): receptor binding domain (RBD), S1/S2 cleavage site (CS), fusion peptide (FP), heptad repeat (HR), and nucleocapsid protein (N).

Pan-coronavirus cross-reactive epitopes, particularly at the conserved regions of the S protein, are associated with antibody response to SARS-CoV-2 (17). Morgenlander et al. defined a set of immunodominant epitopes shared by SARS-CoV-2 as well as endemic coronaviruses and associated them with antibody response to SARS-CoV-2 infection (17). Therefore, to better characterize donor versus endogenously produced antibodies, plasma from 4 recipients (recipients 1–4) was subjected to a high-throughput analysis using a phage-display epitope library, spanning across the SARS-CoV-2 genome as well as the 4 endemic human coronaviruses, SARS, and MERS. Plasma obtained from the recipients before and at 7, 14, and 21–28 days and 2 months after transfusion were assayed. A distinct set of antibodies in the donor plasma could be identified in all recipients, with antibody kinetics similar to those of anti-S and anti-RBD IgG (Figure 3). We also compared the antibody spectrum between adult donors and convalescent response in pediatric recipients who developed endogenous antibody responses. The endogenous antibody responses (in those participants who developed them) represented a distinct set of antibodies, different from those transferred from the donor plasma (Figure 4). Moreover, antibody reactivities of convalescent plasma among adult donors were more diverse and had higher antibody titers, compared with the convalescent response in pediatric recipients, 2 months after transfusion (Figure 4A). Multiple cross-reactive antibodies directed at immunodominant epitopes were transferred from donors to recipients, expanding their antibody repertoire (Figure 4B).

Finally, all febrile recipients (*n* = 7) became afebrile within 3 days of convalescent plasma transfusion or earlier (Supplemental Table 2). Four recipients required intensive care and 3 required noninvasive positive pressure ventilation for up to a week (Supplemental Tables 1 and 4). These recipients also received remdesivir and dexamethasone. In symptomatic recipients for whom data were available, the initial high viral load decreased substantially when measured at day 7 (Supplemental Figure 4). One asymptomatic, negative recipient reported new fever and cough 2 weeks following exposure (recipient 4). This recipient became PCR positive on day 12 and remained positive on day 21 (Supplemental Figure 4).

## Discussion

Currently, only a limited number of case reports and case series have been published on the use of COVID-19 convalescent plasma in children (18–26), and a detailed pharmacokinetic analysis is lacking. In this prospective study, high-titer convalescent plasma appears to be safe when given to high-risk children. Recipient titers 30 minutes after plasma transfusion were about 20-fold lower than donor titers, most likely due to a dilution effect. Despite donor plasma having significant neutralization capacity, only 5%–6% of the donor neutralization titer could be detected in plasma from recipients, even as early as 30 minutes after transfusion.

All participants received a dose of 5 mL/kg COVID-19 convalescent plasma. This dose was informed by previous experience using convalescent plasma for other infections (27), by volume limitations particularly due to inclusion of children with congestive heart disease, and by dosing in adult studies evaluating convalescent plasma, where a similar or lower dose is being utilized (13, 14). Additionally, 1 unit of convalescent plasma (i.e., equivalent to ~3.5 mL/kg) was utilized in a study where high-titer convalescent plasma that was administered early to high-risk older adults decreased subsequent severe respiratory disease (10). This was correlated with an increase in anti-S IgG in recipients, but neutralizing capacity was not measured. Models predicting the relationship between in vitro neutralization levels and observed protection (based on vaccine trials and convalescent cohorts), estimated the neutralization level required for protection against severe disease to be 3% of the mean convalescent level (28). Based on our data, when measured 30 minutes after administration, the recipient will have about 5%–6% of the neutralization capacity of the corresponding donor, which may therefore protect against severe disease. However, this titer is expected to decline and may not provide lasting protection.

While clinical data was collected, this study was not designed to evaluate the clinical efficacy of convalescent plasma. Children who were febrile, became afebrile within 72 hours following administration of convalescent plasma. Five children were hospitalized; 3 developed severe pneumonia before administration of convalescent plasma and ultimately required noninvasive positive pressure ventilation. All of them survived to hospital discharge.

Antibody reactivities of convalescent plasma among adult donors were more diverse, with higher antibody titers, compared with the convalescent response in pediatric recipients, 2 months after transfusion. This is consistent with previous reports demonstrating a lower antibody response in children with COVID-19 (29, 30). Still, children with a more severe COVID-19 course did develop a more robust endogenous antibody response.

Our study has some limitations. First, the sample size of this cohort is small, although we did obtain clinical samples prospectively at several time points for a detailed pharmacokinetic analysis that can be performed adequately, even with a small number of participants (31). Second, some participants in this study were immunocompromised, which could have altered their endogenous antibody responses. Third, single donor plasma was utilized rather than hyperimmune globulin (i.e., pooled high-titer plasma) due to practical issues of producing the product in the settings of a rapid response to a pandemic. Finally, the pharmacokinetic analysis for participants 3, 9, and 10 was limited by the fact they received intravenous immunoglobulins as part of their routine care. Saturation of immunoglobulins with supraphysiological concentrations may lead to accelerated excretion. Indeed, pharmacokinetic analysis showed that transferred IgGs had a shorter half-life in these participants.

To conclude, in this prospective study, administration of high-titer convalescent plasma to high-risk children appears to be safe, with transfer of multiple coronavirus antigen-specific antibodies from the donor to the recipient. Donor plasma had significant neutralization capacity; however, this resulted in only 5%–6% of donor neutralization titer as early as 30 minutes after transfusion and declined thereafter. Achieving higher neutralization titer in recipients will require the use of plasma with much higher neutralizing titers, such as that obtained from individuals who had COVID-19 and were subsequently vaccinated (32). While further research is needed to validate these results in a larger cohort, these data suggest that current use of convalescent plasma in high-risk children may only achieve low titers of neutralizing antibody.

## Methods

### Study design.

The purpose of the study was to evaluate the safety and pharmacokinetics of high-titer COVID-19 convalescent plasma in children (age, 1 month to 18 years) who were defined to be at high-risk for disease progression (Supplemental Figure 1). Briefly, this was based on the American Academy of Pediatrics definition of immunocompromised children and reported high-risk pediatric subpopulations: immunocompromised, hemodynamically significant cardiac disease (e.g., congenital heart disease), lung disease with chronic respiratory failure, and infants, i.e., children at or less than 1 year old (6–8). High-risk children exposed to or infected with SARS-CoV-2 were enrolled to receive convalescent plasma (pretitered >1:320 Euroimmun assay at 5 mL/kg) within 5 days of exposure or 8 days of symptom onset. Exclusion criteria included history of severe allergic reaction to transfusion of blood products and pregnancy. One participant was excluded after receiving the plasma, due to a false-positive SARS-CoV-2 test.

The primary endpoint was safety, defined as the combined cumulative incidence of grade 3 and 4 AEs and serious AEs during the study period. Secondary objectives included the characterization of SARS-CoV-2 antibody pharmacokinetics after plasma administration and evaluation of effects on the natural immunological response to SARS-CoV-2 infection. A total of 38 blood samples and 26 nasopharyngeal swabs were collected and analyzed. Whole blood was collected in acid citrate–dextrose tubes. All samples were deidentified before laboratory testing. The samples were separated into plasma within 12 hours of collection. The plasma samples were immediately frozen at –80°C.

Individuals at or over 18 years of age with a confirmed diagnosis of SARS-CoV-2 by detectable RNA on a nasopharyngeal swab were eligible to donate plasma (33).

### Commercially available enzyme immunoassays.

Plasma specimens were analyzed using the Euroimmun Anti–SARS-CoV-2 IgG and IgA assay targeting the S-1 protein and the Bio-Rad Platelia SARS-CoV-2 total antibody assay targeting the nucleocapsid protein, per the manufacturers’ instructions. The intended use of each test is the qualitative detection of antibodies; however, they also provide semiquantitative output normalized by a calibrator.

### SARS-CoV-2 indirect ELISA.

The ELISA protocol has been detailed previously (33, 34). Briefly, 96-well plates (Immulon 4HBX, Thermo Fisher Scientific) were coated with either full-length S protein or S-RBD at a volume of 50 μL of 2 μg/mL diluted antigen in filtered, sterile 1× PBS (Thermo Fisher Scientific) at 4°C overnight. For serial dilutions of plasma on either S- or S-RBD–coated plates, plasma samples were prepared in 3-fold serial dilutions, starting at 1:20. All assays were performed in duplicate. The following anti-human secondary antibody was used: Fc-specific total IgG HRP (1:5000 dilution, catalog A18823, Invitrogen, Thermo Fisher Scientific). The OD of each plate was read at 490 nm (OD490) on a SpectraMax i3 ELISA Plate Reader (BioTek Instruments). The positive cutoff value for each plate was calculated by summing the average of the negative values and three times the SD of the negative values. All values at or above the cutoff value were considered positive.

### Microneutralization assay.

Plasma neutralizing antibody titers were quantified against 100 fifty percent tissue culture infectious doses (TCID_50_) using a microneutralization assay in VeroE6-TMPRSS2 cells, which has been previously described (33). Briefly, plasma was diluted 1:20 and subsequently 2-fold dilutions were used. Infectious SARS-CoV-2 virus was added to the plasma dilutions at a final concentration of 1 × 10^4^ TCID_50_/mL. After 1-hour incubation at room temperature, 100 μL of each sample dilution was added to 6 wells in a 96-well plate of VeroE6-TMPRSS2 cells and incubated for 6 hours at 37°C. The inocula were removed from the plate, fresh media were added, and the plate was incubated at 37°C for 48 hours. The cells were fixed with 4% formaldehyde (in each well), incubated for 4 hours at room temperature, and stained with Napthol Blue Black (Sigma-Aldrich). We calculated a neutralizing antibody titer AUC value for each sample using the exact number of wells protected from infection at every dilution. Samples with no neutralizing activity were assigned a value of one-half the lowest measured AUC.

### PRNT_50_ and IC_50_.

Vero E6-TMPRSS2 cells were seeded in 6-well plates and allowed to reach 95%–100% confluence. Plasma samples were initially diluted 1:10 and further diluted in 2-folds until 1:1280, with enough volume for duplicates. Equal volumes of virus solution containing 100 PFU of infectious SARS-CoV-2 virus (hCoV-19/USA/WA1/2020) and plasma dilution were mixed and incubated for 1 hour at room temperature. 250 μL of each virus-plasma mixture was added to a seeded well in duplicate and incubated at 37°C for 1 hour, with occasional rocking of the plate to distribute the inoculum evenly. Virus-plasma mixtures were aspirated, and 2 mL of virus overlay media (a mixture of equal parts of 2% methylcellulose in water and 2× MEM containing 10% FBS, 2% Pen/Strep, and 1% L-glutamine) was overlaid on each infected well and incubated for 48 hours at 37°C. The overlay was removed after adding 2 mL/well PBS and cells were fixed with 2 mL/well 4% formaldehyde for at least 12 hours at room temperature. Wells were stained using Napthol Blue Black for at least 6 hours, and plaques were counted to determine PFU and PRNT_50_. IC_50_ values were calculated using the inhibition dose-response, nonlinear regression model in GraphPad Prism 9 software.

### SARS-CoV-2 PCR.

Reverse-transcriptase PCR (RT-PCR) of nasopharyngeal swabs was performed using Center for Disease Control and Prevention (CDC) nCoV N1 and N2 primers to detect the virus. CDC RNase P primer set was used as a control (35). A calibration curve was used to devise a quantitative measure. Viral genome copies are reported. We also report available clinical PCR results performed before plasma administration at the Johns Hopkins clinical microbiology laboratory.

### Programmable phage-display immunoprecipitation and sequencing.

The design and cloning of the 56–amino acid coronavirus libraries as well as phage immunoprecipitation and sequencing is described previously (36). Briefly, 0.2 μL plasma was individually mixed with the coronavirus phage library and immunoprecipitated using protein A– and protein G–coated magnetic beads. A set of 6–8 mock immunoprecipitations (no plasma input) were run on each 96-well plate. Bead washing was implemented on a Bravo liquid handling robot. Magnetic beads were resuspended in PCR master mix and subjected to thermocycling. A second PCR reaction was used for sample barcoding. Amplicons were pooled and sequenced on an Illumina NextSeq 500 instrument. The number of times a clone was detected in each immunoprecipitate was counted and then compared against a negative control without serum (mock immunoprecipitations) using edge-R (R software) and a negative binomial model. Plasma-reactive peptides required at least 100 counts and a 5-fold change from control. Analysis of recipient antibody subsets was performed for antibodies with high fold change at 2 months after plasma transfusion (endogenous antibodies) and for antibodies that were high in the donors and low in the recipients before and 2 months after plasma transfusion (transferred antibodies). Only antibodies unique to each group are shown.

### Pharmacokinetic analysis.

A 1-compartment model with a first-order elimination was used to analyze the time course of the antibody titers using Pumas version 2.0 (Pumas-AI) (37). Clearance and volume of distribution of anti-S IgG and anti-RBD IgG were estimated with an additive residual error. R version 4.0.2 (R Core Team 2020) and RStudio version 1.3.1073 (RStudio Team 2020) were used for graphical analysis.

### Statistics.

Statistical analyses were carried out using GraphPad Prism 8 software. Comparison of continuous variables was performed using (nonparametric) Mann-Whitney *U* test. Statistical significance was accepted at the conventional 2-sided *P* < 0.05 threshold.

### Study approval.

This clinical study (ClinicalTrials.gov NCT04377672) was conducted at the Johns Hopkins Children Center between May 2020 and April 2021 under a US FDA exploratory investigational new drug application (no. 20185). It was approved by the Johns Hopkins Institutional Review Board (IRB00247557) and was conducted according to the ethical standards of the Helsinki Declaration of the World Medical Association.

## Author contributions

OG and SKJ conceived and designed the study. OG, SS, DS, HML, EMB, AART, AAO, EWT, CARB, and SKJ wrote the study protocol. OG, MKB, EWT, MEMY, NPA, and KRC recruited the study participants. AAO and PDJ processed the study samples. SY, KL, ZM, JW, ML, and AP performed PCRs and neutralization assays. AG, CAC, and SLK developed and performed the SARS-CoV-2 antibody ELISAs. EMB and AART provided the COVID-19 convalescent plasma. JVSG and DJ developed the pharmacokinetic model. WRM, SNH, and BL developed and performed the coronavirus VirScan. DS, SS, and AC provided logistical support for the study. OG and SKJ wrote the manuscript with substantial input from all coauthors.

## Supplementary Material

Supplemental data

ICMJE disclosure forms

## Figures and Tables

**Table 1:**
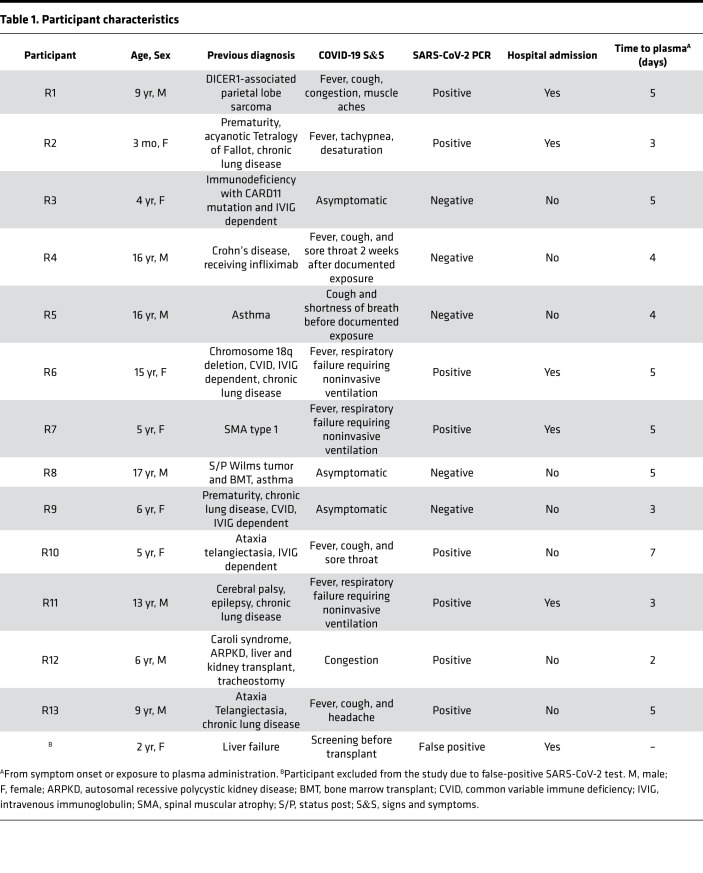
Participant characteristics

## Version 1 12/02/2021

In-Press Preview

## Version 2 01/25/2022

Electronic publication
