# Supplementary material for: Pharmacokinetics of high-titer anti–SARS-CoV-2 human convalescent plasma in high-risk children
Source: JCI Insight. 2022 Jan 25;7(2):e151518. doi: 10.1172/jci.insight.151518 (PMC8855821; doi:10.1172/jci.insight.151518)
Supplement: Supplemental data [file jciinsight-7-151518-s036.pdf]

Pharmacokinetics of High-titer Anti-SARS-CoV-2 Human Convalescent Plasma in High-risk Children

Authors: Oren Gordon[1], Mary Katherine Brosnan[1], SukMin Yoon[2], Dawoon Jung[3], Kirsten Littlefield[2], Abhinaya Ganesan[2], Christopher A. Caputo[2], Maggie Li[2], William R. Morgenlander[4], Stephanie Henson[4], Alvaro A. Ordonez[1], Trisha De Jesus[1], Elizabeth W. Tucker[5], Nadine Peart Akindele[1], Zexu Ma[2], Jo Wilson[2], Camilo A. Ruiz-Bedoya[1], M. Elizabeth M. Younger[6], Evan M. Bloch[7], Shmuel Shoham[8], David Sullivan[2], Aaron A. R. Tobian[7], Kenneth Cooke[9], Ben Larman[4], Jogarao V.S. Gobburu[3], Arturo Casadevall[2], Andrew Pekosz[2], Howard Lederman[6], Sabra L. Klein[2] and Sanjay K. Jain[1*]

Affiliations:
[1] Division of Infectious Diseases, Department of Pediatrics, Johns Hopkins University School of Medicine, Baltimore, MD, USA.
[2] W. Harry Feinstone Department of Molecular Microbiology and Immunology, Johns Hopkins University Bloomberg School of Public Health, Baltimore, MD, USA
[3] Center for Translational Medicine, University of Maryland School of Pharmacy, Baltimore, MD, USA.
[4] Division of Immunology, Department of Pathology, Johns Hopkins University School of Medicine, Baltimore, MD, USA
[5] Department of Anesthesiology and Critical Care Medicine, Johns Hopkins University School of Medicine, Baltimore, MD, USA.
[6] Division of Immunology, Department of Pediatrics, Johns Hopkins University School of Medicine, Baltimore, MD, USA.
[7] Division of Transfusion Medicine, Department of Pathology, Johns Hopkins School of Medicine, Baltimore, MD, USA
[8] Division of Infectious Diseases, Department of Medicine, Johns Hopkins University School of Medicine, Baltimore, MD, USA
[9] Division of Oncology, Department of Pediatrics, Johns Hopkins University School of Medicine, Baltimore, MD, USA.

*To whom correspondence should be addressed: 1550 Orleans Street, CRB-II, Rm 1.09, Baltimore, MD, 21287. Phone 410-502-8241. sjain5@jhmi.edu (S.K.J.)

Supplemental Table 1. Study participants (recipients).

#	Sex	Age	Race / Ethnicity	Co-morbidities	Weight (kg)	Height / length (cm)	Time to plasma [1] (days)	Recipient / Donor Blood type	Plasma dose (mL/kg)
**1**	M	9y	Hispanic	DICER1-associated parietal lobe sarcoma	44.6	141	5	O+/O+	5
**2**	F	3m	AA	Prematurity, acyanotic Tetralogy of Fallot, chronic lung disease, planned for corrective heart surgery	4.5	53	3	B+/B+	5
**3**	F	4y	Asian	Immunodeficiency associated with CARD 11 mutation, IVIG[2] dependent	13.8	98	5	O+/O+	5
**4**	M	16y	White	Crohn's disease, receiving infliximab	64.3	172.9	4	A-/A-	4
**5**	M	16y	AA	Asthma	65.8	174	4	O+/O+	4
**6**	F	15y	White	Chromosome 18q deletion, CVID, IVIG[2] dependent, chronic lung disease	25.4	135.9	5	O+/O+	5
**7**	F	5y	White	SMA type 1	12.5	88	5	O+/O+	5
**8**	M	17y	White	S/P Wilms tumor and BMT, asthma	49.1	162.5	5	A+/A+	4
**9**	F	6y	White	Prematurity, chronic lung disease, CVID, IVIG[2] dependent	16.4	106.9	3	O+/O+	5
**10**	F	5y	White	Ataxia telangiectasia, IVIG[2] dependent	19.1	102	7	O+/O+	5
**11**	M	13y	White	Cerebral palsy, epilepsy, pulmonary insufficiency	41.7	144.8	3	A+/A-	5
**12**	M	6y	AA	Caroli syndrome, ARPKD, liver and kidney transplant, tracheostomy	14.1	90	2	A+/A+	5
**13**	M	9y	White	Ataxia Telangiectasia, chronic lung disease	31.5	-	5	O+/O+	5

[1]Time from symptom onset / exposure to administration of COVID-19 convalescent plasma. [2]P#3 - received IVIG, 10 days before convalescent plasma and then every 3 weeks; P#6 - received SubQ-IG, 4 days before convalescent plasma and then every 7 days; P#9 - received SubQ-IG, 4 days before convalescent plasma and then every 7 days; P#10 - received SubQ-IG, 5 days before convalescent plasma and then every 7 days. F - Female; M - male; y - years; m - months; AA - African American; ARPKD - autosomal recessive polycystic kidney disease; BMT - bone marrow transplant; CVID - common variable immune deficiency; IVIG - intravenous immunoglobulin; SMA - spinal muscular atrophy.

Supplemental Table 2. Clinical presentation at study entry.

Recipient #	Infected children[1]	1	2	3	4	5	6	7	8	9	10	11	12	13
SARS-CoV-2 NP RT-PCR	8 (100%)	Pos	Pos[2]	Neg	Neg	Neg	Pos	Pos	Neg	Neg	Pos	Pos	Pos	Pos
Fever	8 (100%)	Y	Y	N	N	N	Y	Y	N	N	Y	Y	N	Y
Duration of fever (days)	3 days	3	3	NA	NA	NA	5	5	NA	NA	1	1	NA	2
Cough	6 (75%)	Y	N	N	N	Y	Y	Y	N	N	Y	Y	N	Y
Shortness of breath	4 (50%)	N	Y	N	N	Y	Y	Y	N	N	N	Y	N	N
Congestion and/or runny nose	3 (37.5%)	Y	N	N	N	Y	Y	N	N	N	Y	Y	Y	Y
Sore throat	1 (12.5%)	N	NA	N	N	Y	NA	N	N	N	Y	NA	N	N
Diarrhea	1 (12.5%)	N	N	N	N	N	N	N	N	N	Y	N	N	N
Vomiting	1 (12.5%)	N	N	N	N	N	N	Y	N	N	N	N	N	N
Headache	1 (12.5%)	N	NA	N	N	Y	NA	N	N	N	N	NA	N	Y
Rash	0 (0%)	N	N	N	N	N	N	N	N	N	N	N	N	N
Muscle aches	2 (25%)	Y	NA	N	N	N	NA	N	N	N	N	NA	N	Y
Loss of smell or taste	0 (0%)	N	NA	N	N	N	NA	N	N	N	N	NA	N	N
Hospitalization	5 (62.5%)	Y	Y	N	N	N	Y	Y	N	N	N	Y	N	N
Length of admission (days)	7 days	3	14	NA	NA	NA	4	7	NA	NA	NA	23	NA	NA
Admission to PICU	4 (50%)	N	Y	N	N	N	Y	Y	N	N	N	Y	N	N
Length of admission to PICU (days)	5 days	NA	4	NA	NA	NA	4	6	NA	NA	NA	6	NA	NA
Need for supplemental oxygen	4 (50%)	N	Y	N	N	N	Y	Y	N	N	N	Y	N	N
Need for non-invasive positive pressure ventilation	3 (37.5%)	N	N	N	N	N	Y	Y	N	N	N	Y	N	N
Other therapy for COVID-19	3 (37.5%)	N	N	N	N	N	Y[3]	Y[3]	N	N	N	Y[3]	N	N

[1]Children with positive SARS-CoV-2 PCR; Testing performed using research and/or clinically approved tests; [2]Positive by clinically approved test only. N (%) or median days are presented. [3]Remdesivir and dexamethasone. PICU – Pediatric Intensive Care Unit; NA - non-applicable.

Supplemental Table 3. Laboratory studies at presentation.

Recipient #	1	2	3	4	5	6	7	8	9	10	11	12	13
WBC (k/mm^3)	2	12.41	28	5.28	4.78	18.78	13.08	6.82	5.12	3.74	8.18	8.78	4.69
HGB (g/dL)	6.6	15.2	10	14.2	15	14.6	13	14.6	10.5	13.1	15.2	12.8	14.4
Platelets (k/mm^3)	46	321	184	208	309	264	361	288	251	225	166	526	292
Neutrophil Count (k/mm^3)	1.82	4.59	2.25	2.65	2.1	18.02	8.41	3.6	1.05	2.22	6.88	4.32	2.23
Lymphocyte Count (k/mm^3)	0.04	6.58	25.59	1.94	1.79	0.25	3.94	2.17	3.59	1.04	0.71	3.75	1.51
Sodium (mEq/L)	136	137	138	139	141	134	133	140	140	140	146	137	141
BUN (mg/dL)	11	10	10	14	16	6	3	17	14	12	9	8	7
Serum Creatinine (mg/dL)	0.5	0.2	0.2	0.6	0.9	0.3	0.2	1.1	0.3	0.3	0.4	0.3	0.3
ALT (U/L)	35	14	13	18	17	27	18	21	13	31	13	19	51
CRP (mg/dL)	NA	<0.1	NA	NA	NA	9.1	1.2	NA	NA	NA	5.3	NA	NA

ALT – alanine aminotransferase test; BUN – blood urea nitrogen; CRP – c-reactive protein; HGB – hemoglobin; NA – non-applicable; WBC – white blood cell count.

Supplemental Table 4. Follow up evaluation 7 to 60 days post-transfusion of convalescent plasma.

Recipient #	1	2	3	4	5	6	7	8	9	10	11	12	13
**Fever**	N	N	N	Day 10-12	N	N	N	N	N	N	N	N	N
**Cough**	Until day 9	N	N	Day 10-12	N	N	N	N	N	Until day 10	Y	N	N
**Shortness of breath**	N	N	N	N	N	Resolved at day7	N	N	N	N	Y	N	N
**Congestion and/or runny nose**	N	N	N	N	N	N	N	N	N	Y	Y	Y	N
**Sore throat**	N	N	N	Day 10-12	N	NA	N	N	N	N	NA	N	N
**Diarrhea**	N	N	N	N	N	N	N	N	N	N	N	N	N
**Vomiting**	N	N	N	N	N	N	N	N	N	N	N	N	N
**Headache**	Days 7-11	NA	N	N	N	NA	N	N	N	N	NA	N	N
**Rash**	N	N	N	N	N	N	N	N	N	N	N	N	N
**Muscle aches**	N	NA	N	N	N	NA	N	N	N	N	NA	N	N
**Loss of smell or taste**	Until day 11	NA	N	N	N	NA	N	N	N	N	NA	N	N
**COVID related hospitalization**	N	N	N	N	N	Until day 7	N	N	N	N	Until day 23	N	N
**Need for supplemental oxygen**	N	N	N	N	N	Until day 7	N	N	N	N	Until day 15	N	N
**Need for non-invasive positive pressure ventilation**	N	N	N	N	N	Until day 5	Back to baseline at day 7	N	N	N	Until day 6	N	N
**[1]SARS-CoV-2 NP RT-PCR**	Neg at day 14	Neg at day 7	Neg	Pos at day 12 and 21	N	Missing data	Neg at day 34	N					

[1]See Supplemental Figure 1B for additional details. NA – non-applicable.

Between 1 month and 18 years of age at the time of consent

AND

Immunocompromised[1]

OR

Medically complex children[2]

OR

Hemodynamically significant cardiac disease[3]

OR

Lung disease with chronic respiratory failure[4]

OR

Obesity[5]

OR

Infant, i.e. child ≤1-year old[6,7]

AND

Confirmed infection: Child who tested positive for COVID-19 and is no more than 7 days after onset of symptoms (and within 8 days at the time of receipt of plasma).

OR

High-risk exposure[8]

- Household member or daycare center (same room) exposure
- Negative for SARS-CoV-2 (nasopharyngeal swab)
- Exposure within 4 days prior to enrollment (and within 5 days at the time of receipt of plasma)

[1]Primary or acquired immunodeficiency e.g. recipient of a bone marrow or solid organ transplant in the last 12 months (or at any time if concurrent with graft-versus host disease), recipient of chemotherapy for a malignancy within the past 6 months, HIV with CD4 (<30% for ≤12 months old; <25% for 12–35 months; <20% for 36–59 months or <350 for all other ages), receiving immunosuppressive or immunomodulatory treatments (e.g., high-dose steroids [≥2 mg/kg/day of systemic prednisone or equivalent for ≥14 days], tacrolimus, sirolimus, cyclosporine, antithymocyte globulin - ATG, mycophenolate, methotrexate, etc.)

[2]Defined as children who has long-term dependence on technological support (including tracheotomy) associated with developmental delay and/or genetic anomalies (Shekerdemian LS, Mahmood NR, Wolfe KK, et al. Characteristics and Outcomes of Children With Coronavirus Disease 2019 (COVID-19) Infection Admitted to US and Canadian Pediatric Intensive Care Units. *JAMA Pediatrics* 2020

[3]Hemodynamically significant cardiac disease (e.g., congenital heart disease)

[4]Lung disease with chronic respiratory failure (e.g., patients with asthma, cystic fibrosis, bronchiectasis, chronic lung disease of prematurity, tracheostomy / ventilator dependency, restrictive lung disease, severe neuromuscular disease, etc.)

[5]Defined as a body mass index (BMI) ≥95th percentile for children and teens of the same age and sex (CDC. "Defining Childhood Obesity" https://www.cdc.gov/obesity/childhood/defining.html. 2018; 3 July. CDC. "People with Certain Medical Conditions" https://www.cdc.gov/coronavirus/2019-ncov/need-extra-precautions/people-with-medical-conditions.html 2020; 30 July).

[6]CDC. Coronavirus Disease 2019 in Children — United States, February 12–April 2, 2020. *https://wwwcdcgov/mmwr/volumes/69/wr/mm6914e4htm?s_cid=mm6914e4_e&deliveryName=USCDC_921-DM25115* 2020.

[7]Dong Y, Mo X, Hu Y, et al. Epidemiological Characteristics of 2143 Pediatric Patients With 2019 Coronavirus Disease in China. *Pediatrics* 2020: e20200702.

[8]All criteria below should be met

Supplementary Figure 1. Eligibility criteria

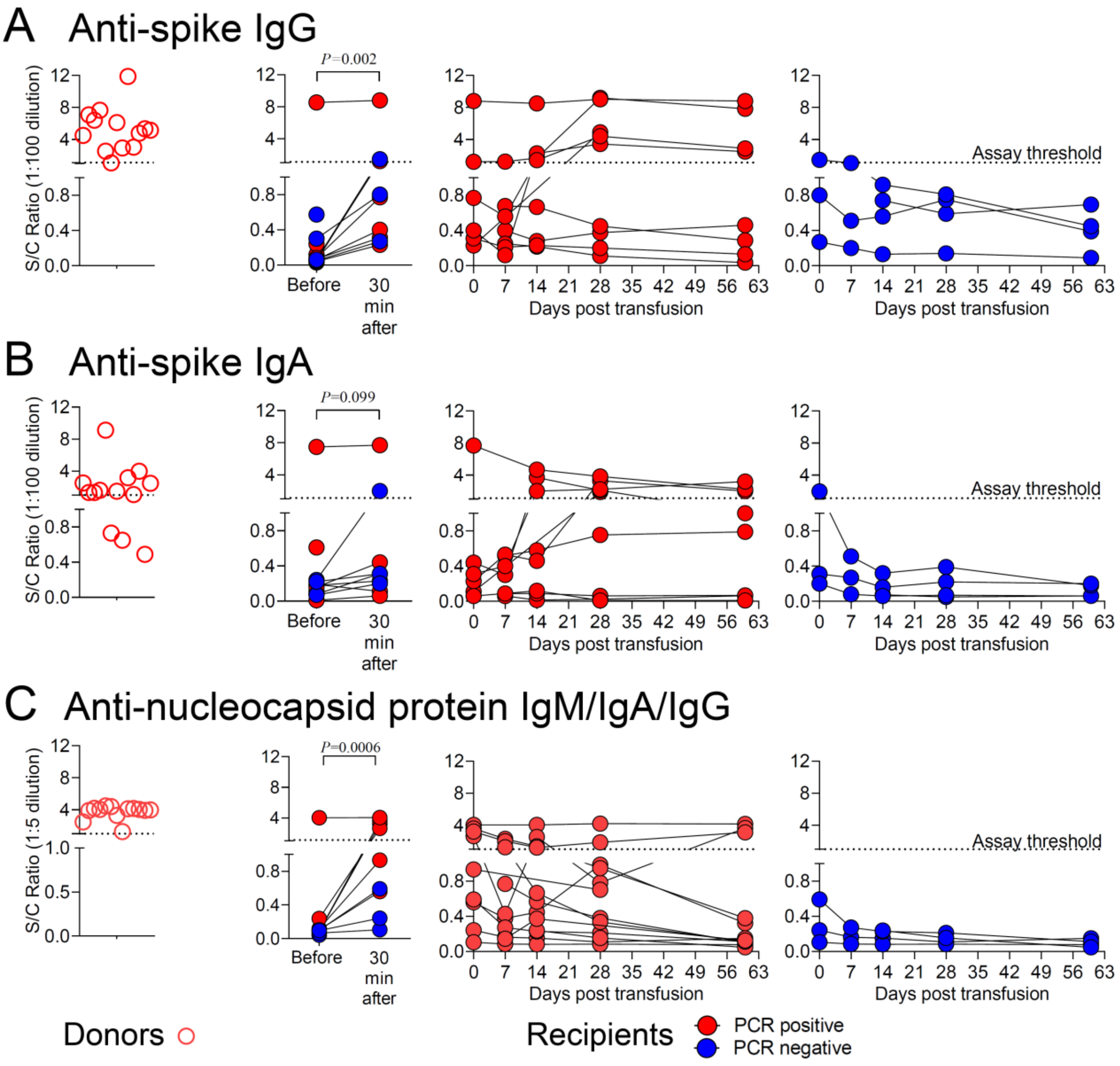

Supplementary Figure 2. Antibody titers in donors and recipient plasma measured by commercial assays. Results presented as signal to cut-off ratio (S/C). Recipients are graphed separately based on SARS-CoV-2 positivity, with those PCR negative (blue) on far right. Anti-spike IgG (**A**) and IgA (**B**) antibodies measured by ELISA (Euroimmun, Lubeck, Germany). Y-axes are segmented to demonstrate the manufacturer recommended assay thresholds: negative (S/C ratio < 0.8), borderline, (S/C ratio ≥0.8 & <1.1) or positive (S/C ratio ≥1.1). (**C**) Anti-nucleocapsid (N) IgM/IgA/IgG measured using EIA (BioRad Platelia, Marnes-la-Coquette, France). Y-axes are segmented to demonstrate the manufacturer recommended assay thresholds: negative (S/C ratio < 0.8), borderline, (S/C ratio ≥0.8 & <1.0) or positive (S/C ratio ≥1.0). For all assays, n=13 pairs of donors and recipients. A two-tailed Mann-Whitney U test was used to compare donor to recipient titers and recipient titers before and 30 minutes after transfusion. The corresponding *p*-values are shown.

A Summary of PK analysis

	Weight (kg)	Anti-Spike IgG		Anti-RBD IgG	
		CL (AUC/day)	$T_{1/2}$ (days)	CL (AUC/day)	$T_{1/2}$ (days)
R2	4.49	1.05	22.9	0.74	21.5
R3	13.8	0.77	7.3	1.1	9.2
R4	64.3	1.68	18.6	0.98	13
R5	65.8	1.73	6.3	2.05	7.6
R8	49.1	0.86	15.5	0.73	34.3
R9	16.4	3.24	17.2	0.49	10.1
R10	19.1	0.67	17.7	0.49	11.8

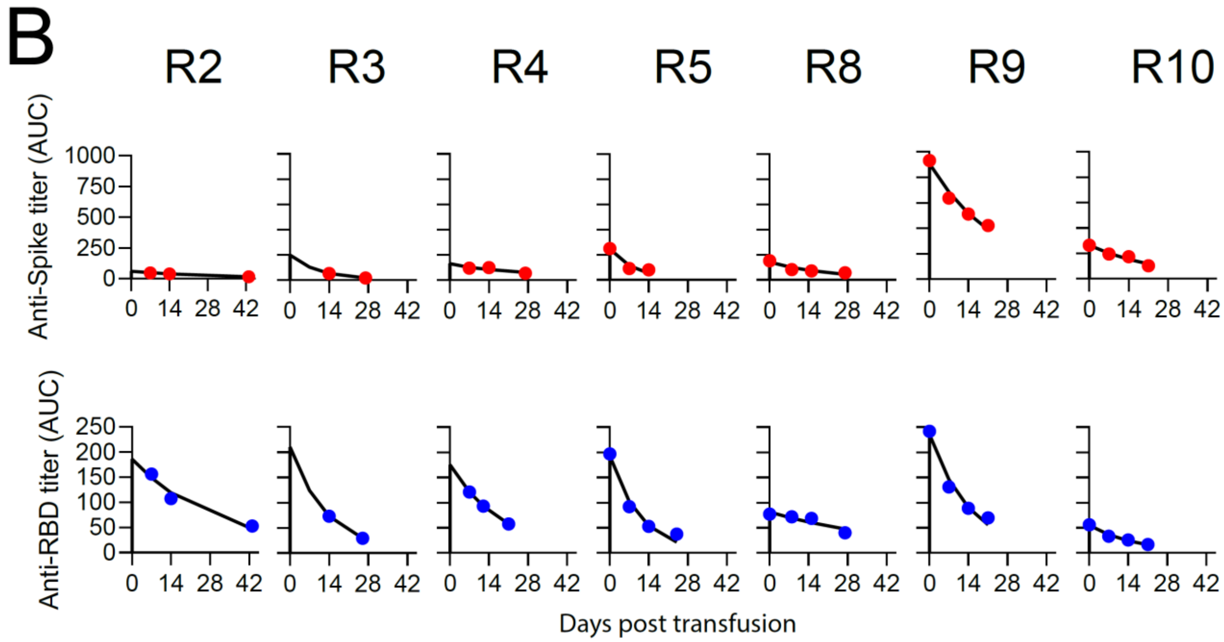

Supplementary Figure 3. Pharmacokinetic (PK) analysis of antibody titers in recipients. (A) Model predicted PK characteristics. Clearance (CL) and half-life ($T_{½}$) of anti-spike and anti-RBD IgG were estimated using two stage analysis. (**B**) Individual predictions of anti-spike and anti-RBD IgG from PK analysis. Red and blue dots represent the observed titers and black lines present the predicted time course of titers.

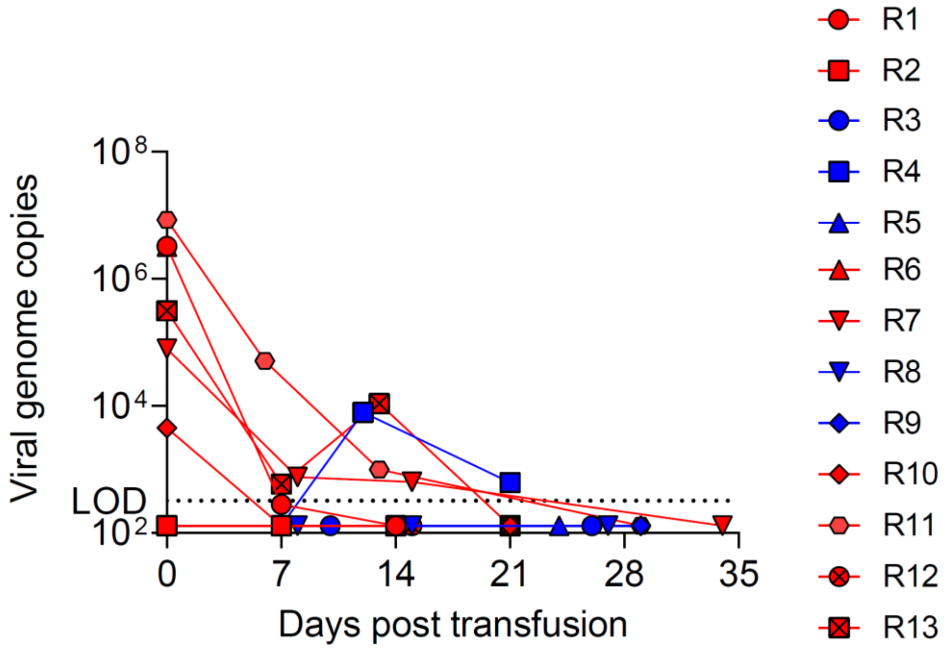

Supplementary Figure 4. Quantitative SARS-CoV-2 PCR. Nasopharyngeal swabs were taken on the day of and weekly following plasma administration for up to 4-5 weeks. Viral genome copies are shown for each recipient (red for PCR-positive and blue for PCR-negative). For recipients R2 and R12, a clinical (non-quantitative) SARS-CoV-2 PCR was positive at day 0. LOD – limit of detection.
